# Development and Testing of an Out‐of‐School Hours Care Professional Development Program: A Pilot Cluster Randomised Controlled Trial

**DOI:** 10.1002/hpja.70056

**Published:** 2025-06-10

**Authors:** Andrew J. Woods, Yasmine C. Probst, Jennifer Norman, Karen Wardle, Sarah T. Ryan, Linda Patel, Ruth K. Crowe, Megan Hammersley, Kurt Morton, Rebecca M. Stanley, Lauren Taylor, Anthony D. Okely

**Affiliations:** ^1^ School of Health and Society, Faculty of the Arts, Social Sciences and Humanities University of Wollongong Wollongong New South Wales Australia; ^2^ Early Start, Faculty of the Arts, Social Sciences and Humanities University of Wollongong Wollongong New South Wales Australia; ^3^ School of Medical, Indigenous and Health Sciences, Faculty of Science Medicine and Health University of Wollongong Wollongong New South Wales Australia; ^4^ Health Promotion Service Illawarra Shoalhaven Local Health District Warrawong New South Wales Australia; ^5^ Health Promotion Service South Western Sydney Local Health District Liverpool New South Wales Australia; ^6^ Centre for Population Health NSW Ministry of Health St Leonards New South Wales Australia

**Keywords:** child, child care, healthy eating, physical activity, primary school, randomised controlled trial, staff development

## Abstract

**Background:**

The aim of this study was to describe the co‐creation and test the feasibility, acceptability and potential efficacy of an Out of School Hours Care (OSHC) staff professional development intervention to increase child adherence to moderate‐to‐vigorous‐intensity physical activity (MVPA) guidelines and fruit and vegetable (F&V) provision in the OSHC setting.

**Methods:**

Four OSHC services participated in a 1‐month, 2‐arm pilot cluster randomised controlled trial. Services had their before and after school care programmes visited twice at baseline and at follow‐up. Feasibility and acceptability were assessed through recruitment/attendance rates and online feedback surveys. Child MVPA was assessed using Actigraph accelerometers and F&V provision with direct observation.

**Results:**

Intervention adherence was feasible, with 60% of total staff employed by services completing the intervention. The intervention was acceptable, with all participants agreeing to the intervention being enjoyable and improving their knowledge of physical activity (PA) and healthy eating. The proportion of children meeting guidelines of 15 min of MVPA in before school care and 30 min in after school care increased over time in both groups. There were no significant between‐group differences in these changes; however, small to medium effect sizes were detected. Frequency of fruit provision increased more in before school care intervention programmes and vegetable provision increased more in both before and after school care intervention programmes.

**Conclusions:**

A PA and healthy eating professional development intervention in OSHC is both feasible and acceptable.

**So What?:**

Larger trials are recommended to evaluate intervention scale‐up and effectiveness on child MVPA and service F&V provision.

## 1 Introduction

Physical activity (PA) and healthy eating are critical to support children's health and wellbeing, with both key components of the Australian Government's National Action Plan for the Health of Children and Young People [1]. Moderate‐to‐vigorous‐intensity physical activity (MVPA) is, particularly, important for school‐aged children and youth, with relationships between MVPA and health outcomes such as physical fitness and cardiometabolic biomarkers being stronger than lower intensity activity levels [2]. MVPA has also been linked to reduced symptoms of depression and anxiety in youth [3]. For healthy eating, fruit and vegetable consumption is important for several health outcomes such as the prevention of cardiovascular disease and depression [4]. Despite these health benefits, a large proportion of Australian children are not meeting national PA and nutrition guidelines. Less than one‐quarter (23%) of children aged 5–12 years from New South Wales, Australia meet the recommended 60 min of MVPA per day [5]. Among children aged 4–8 years, 29% and 98% fall short of meeting the recommended intakes of fruit and vegetables, respectively. For children aged 9–11 years, 35% and 97% are not meeting these recommendations [6].

Out of School Hours Care (OSHC) has been identified as a priority setting for improving dietary habits and PA levels among Australian children [7]. Australian OSHC services offer supervision and care to children aged 5–12 years before and after school, and during school holiday periods (vacation care). It is a widely utilised setting with over 4900 OSHC services operating across Australia, with more than half a million children attending regularly in 2023 [8]. The Australian OSHC PA guidelines, which were recently drafted, recommend scheduling PA opportunities for 45 min during before school care and 90 min during after school care to assist children to achieve 15 min (25% of total daily guideline) and 30 min (50% of total daily guideline) of MVPA in these respective periods [9].

There are no specific nutrition guidelines for OSHC; however, the national governing body, Australian Children's Education and Care Quality Authority (ACECQA) directs services to adhere to the Australian Dietary Guidelines [10, 11]. Nutrition Australia, which is Australia's peak community nutrition education body, also encourages fruit and vegetables to be provided daily in before‐school care and strongly recommends that they be provided daily in after‐school care [12]. Despite this, previous studies have investigated the PA and food environments of Australian before‐and after‐school care services and identified low adherence to MVPA recommendations and a need for increased fruit and vegetables provision [13, 14, 15]. These studies highlighted staff health‐promoting behaviours, activity context, training and policies as factors which influence this in OSHC.

Studies conducted in the United States have identified professional development of OSHC staff as having the potential to increase children's MVPA and service provision of fruit and vegetables [16, 17]. A recent Australian study of before school care found that services are more likely to provide vegetables if their staff had received training in healthy eating [18]. Another study identified face‐to‐face training for Australian OSHC staff improved their confidence to provide a health promoting environment [19]. Despite these findings, to the authors' knowledge, there are few studies investigating the effect that a professional development programme for staff can have on these environments in Australian OSHC services. An Australian study by Veldman et al. [20] involved a professional development component; however, the focus was PA and executive function, and it was only tested in after school care.

The aim of this current study, therefore, was to (1) describe the co‐creation of an OSHC staff professional development programme to promote PA and healthy eating and (2) investigate the feasibility, acceptability and potential efficacy of this programme to increase child adherence to MVPA guidelines and fruit and vegetable provision in the before‐andand‐after school care of OSHC services located in New South Wales, Australia. It was hypothesised that (1) the proportion of children obtaining ≥ 15 min of MVPA in before school care and ≥ 30 min of MVPA in after school care would increase more in intervention services compared to control services; (2) the frequency of observation days fruit and vegetables were provided in before and after school care would increase more in intervention services compared to control and (3) the professional development programme would see high attendance and adherence rates and have high acceptability among OSHC staff in the intervention group.

## 2 Methods

### 2.1 Intervention Development

The OSHC professional development intervention presented in this study was guided by a framework for utilising participatory methodologies in the co‐creation of public health interventions developed by Leask et al. [21]. This framework involves four stages of co‐creation principles: planning, conducting, evaluating, and reporting. The intervention mode was not pre‐determined, with co‐creators able to contribute to what the intervention would look like. The development process is described according to these stages in Supporting Information Intervention Development. The intervention co‐creation was approved by the University of Wollongong Human Research Ethics Committee (2021/ETH11747).

### 2.2 Trial Design

The ENHANCE OSHC intervention was tested for feasibility, acceptability and potential efficacy using a pilot cluster parallel‐group wait‐listed RCT. The reporting of this study follows the CONSORT statement for cluster RCTs and the extension for pilot and feasibility trials [22, 23]. The intervention study was approved by the University of Wollongong Human Research Ethics Committee (2023/ETH00140) and registered prospectively with the Australian New Zealand Clinical Trials Registry (ACTRN12623000176662p). This study is evaluating the 3‐h in‐person training session and ongoing support phone calls and text messages. A full evaluation of the Eat Smart Play Smart (ESPS) app utilised in the intervention is currently underway in a separate study (Patel et al. unpublished), so is not being evaluated here.

### 2.3 Recruitment, Participants, and Randomisation

As the study was a pilot, the sample size was not designed to detect statistically significant differences between intervention and control services. A recruitment target of six OSHC services was initially established; however, following lower than expected uptake aligned with timeline constraints, this was reduced to four services. To be eligible, services were required to enrol 10 or more children per morning and afternoon, operate both before and after school, provide food and not exclusively advertise themselves as a homework or PA club. Half of all services located in the Illawarra Shoalhaven Local Health District and South Western Sydney Local Health District who met eligibility criteria were contacted via email and invited to participate (*n* = 176). As recruitment for this study occurred at the same time as another OSHC study, eligible services in these Local Health Districts were randomly assigned to each of the studies to avoid overburdening participants during recruitment. This was performed in a 1:1 ratio using a computerised random number generator (http://www.random.org). Recruitment phone calls were made to 50 services to assist recruitment, with voicemails left when there was no answer.

Following written director consent, participating services distributed information sheets and consent forms to the staff and parents/guardians of children from their service to assist with staff and child recruitment. Staff information sheets included detail on being invited to a 3‐h in‐person training session and sharing their mobile phone number for delivery of the weekly text messages. Parent/guardian information sheets included detail on their child being invited to wear an accelerometer during data collection visits at their centre. To further assist with child recruitment, visits were made to participating services by the lead researcher to distribute study information in person to parents upon child drop‐off and collection.

Random allocation to intervention and waitlist‐control groups occurred at the conclusion of baseline data collection. Randomization was performed in a 1:1 ratio using a computerised random number generator (http://www.randomization.com). An independent data manager conducted the randomisation. The researcher responsible for implementing the intervention was the only person informed about group allocation. This researcher did not conduct follow‐up data collection due to being unblinded to group allocations.

### 2.4 Intervention Overview—ENHANCE OSHC

The co‐created professional development programme was named ‘ENHANCE Out of School Hours Care’ (ENHANCE OSHC). ENHANCE stands for ‘Evidence‐based Healthy Activity and Nutrition for Children and Environments’, the name of the University and Government research group that oversaw this research. The programme involved a 3 h in‐person training session for OSHC staff. The first half of the session focussed on healthy eating topics, with the second half covering PA. Healthy eating and PA education was provided including practical activities to develop skills for promoting healthy behaviours in children attending OSHC. An overview of the session structure can be found in the Supporting Information Training Session Structure. The possibility of online training was considered during intervention development; however, co‐creators expressed a preference for in‐person training. This is supported in a study by Lee et al. [24] which found that an OSHC PA and healthy eating professional development intervention was more effective when offered in‐person than online.

During the training session, staff from each OSHC service were asked to create their own service action plans, one for both PA and healthy eating (Supporting Information_PA Action Plan and Supporting Information_Healthy Eating Action Plan). They were asked to create goals using the SMART framework [25] which could be achieved over a 4‐week period. A copy of these plans was collected, and participants received ongoing support during the intervention period. The 4‐week timeline allowed the intervention to be completed over one school term and coincide with study deadlines. The ongoing support involved once weekly health‐promoting text messages to all participants and once weekly phone calls with each service director. The information in the text messages and phone call discussions focussed on goals created in the action plans of each service.

Intervention services were also provided access to the ESPS smartphone application (app) which was co‐created with OSHC staff as part of a separate study and is being fully evaluated by researchers within the ENHANCE OSHC working group (Patel et al. unpublished). The content of the app draws on information from the ESPS manual and other relevant sources. The app was used in this study to provide supplementary evidence‐based PA and healthy eating information and practical tools. It includes features designed to assist staff with necessary tasks such as developing PA schedules, creating or revising service menus and updating service policies. The app provides an extensive selection of PA videos and recipes for OSHC educators to easily implement. Participants were asked to use the ESPS app over the 4‐week intervention period for activity, recipe and policy ideas in the service.

### 2.5 Procedures

Between May and June 2023, each participating service's before and after school programmes were visited by trained data collectors on two unannounced, non‐consecutive occasions to conduct baseline measurements. Following randomisation, consenting staff from intervention services were invited to attend a 3‐h ENHANCE OSHC training session held at a mutually accessible location. The training session was delivered by the lead researcher with experience in health promotion, with the intervention period commencing immediately after. Participants were given access to the app during the session and were sent a text message once per week during the 4‐week intervention period. A download link for the app was also provided to services, allowing staff who did not attend the training to utilise the app. The directors of each service also received a weekly phone call from a researcher on the study. Staff were asked to complete an online survey at the end of the training and the end of the intervention period. Once the intervention period concluded, follow‐up data collection was conducted immediately after, occurring between August and September 2023. Data collectors were blinded to group allocation. Control services were wait‐listed and did not receive the intervention until the conclusion of follow‐up data collection. Study data were collected and managed using the Research Electronic Data Capture tool (REDCap) [26, 27], including the REDCap Mobile Application, hosted at the University of Wollongong.

### 2.6 Feasibility and Acceptability Measurements

Feasibility of the intervention was assessed by analysing OSHC service recruitment numbers, OSHC staff recruitment numbers and attendance rates of consenting participants at the training session. Acceptability of the intervention was assessed using two online feedback surveys completed by staff participants. The first survey was completed at the end of the in‐person training session and consisted of eight questions asking participants to rate their enjoyment, knowledge and skills gained during the session using a 5‐point Likert scale. The second survey was completed at the end of the 4‐week intervention period and asked seven questions regarding the usefulness of the ongoing support and intervention as a whole. The questions were a mixture of 5‐point Likert scale, multiple choice and short answer. Weekly ongoing support phone calls to directors also provided further information on acceptability, as conversations provided participants opportunities to comment on the intervention. Participant usage of the ESPS app was also monitored, including app downloads, number of app sessions, average length of app sessions and day of the week the app sessions occurred.

### 2.7 Intervention Adherence

Adherence to the intervention during the 4‐week period was monitored qualitatively with the weekly ongoing support phone calls to directors. During these calls, the director was asked about the implementation of their service action plans and use of the app among staff.

### 2.8 Potential Efficacy Outcomes and Measurements

The primary potential efficacy outcomes of this study were (1) the frequency of provision of fruit and vegetables in before school and vegetables in after school care and (2) the proportion of children who participated in at least 15 min of MVPA while attending before school care and at least 30 min of MVPA while attending after school care. The secondary potential efficacy outcomes were staff engagement in promotion of PA and healthy eating behaviours.

The measurements used to test the potential efficacy of the intervention have been used previously in cross‐sectional studies exploring the PA and healthy eating environment of Australian before and after school care services [13, 14, 15]. These measures have been discussed in detail elsewhere [7], and will be briefly described below. All potential efficacy measures were conducted on intervention and control services at baseline and follow‐up.

### 2.9 Child PA and Sedentariness

Child PA and sedentariness were measured using ActiGraph GT3X+ accelerometers (ActiGraph Corporation, Pensacola, FL) initialised with a sampling rate of 30 Hz. Accelerometers were placed on the right hip of children with parental consent as they arrived at the service and their sex, school grade and time on of wear were recorded. ActiGraphs were collected as children departed the service and time off of wear was recorded. A day of valid accelerometry was defined as a child wearing the accelerometer for at least 30 min in before school care and 60 min in after school care. A threshold of 60 min is commonly used in studies of after school care [14, 28]; and a recent study in before school care found mean attendance time approximately half of that in after school care; therefore, a threshold of 30 min was used [13]. Children whose accelerometer wear time did not meet the required minutes were excluded from data analysis.

### 2.10 Staff Physical Activity Promotion Behaviours

The validated time sampling tool, System for Observing Staff Promotion of Physical Activity and Nutrition (SOSPAN) [29] was used to capture staff PA promotion behaviour. This tool was created for use in after school programmes in the United States and is commonly used for observing Australian OSHC environments [14, 30]. During data collection, data collectors moved between zones of an OSHC service scanning from left to right and checking off pre‐defined items they were observing. Staff PA promotion behaviours captured included staff engaging in PA, staff verbally promoting PA, and staff discouraging PA.

### 2.11 Food and Beverage Provision and Staff Nutrition Promotion Behaviours

Food and beverages provided by the service for breakfast (before school care) and afternoon snack (after school care) were captured by direct observation using a modified version of a food observation tool created by Kelly et al. [31]. Foods and beverages were recorded into subgroups which informed categorisation into the five food groups of the Australian Dietary Guidelines [10], with additional groups for discretionary foods and beverages. Categorisation was conducted by the lead author (AW) and guided by the Australian Health Survey food classification system [32] and the Australian Health Survey Discretionary Food List [33]. As food had been previously entered by trained data collectors a priori into categories of the food audit tool, one researcher was deemed sufficient for this. Photographs were also taken of the food provided, as well as the packing and nutrition labels to assist with categorisation. A food and beverage environmental context form was also completed each visit during breakfast (before school care) and afternoon snack (after school care) to capture information on staff behaviours during mealtime. These behaviours include staff verbally promoting healthy eating and staff verbally discouraging healthy eating.

### 2.12 Statistical Analysis

Feasibility and acceptability of the intervention were reported using percentages, and summarising qualitative data from the ongoing support phone calls and online feedback surveys. As the number of food observation days were insufficient for comparative analysis, changes in provision of food groups within control and intervention services were reported descriptively. Accelerometry data were downloaded in 15‐s epochs from ActiLife software (ActGraph Corporation, Pensacola, FL, USA), and PA intensities calculated using Evenson cut‐points [34]. Analyses were conducted using Jamovi software (v2.3, The Jamovi project, Sydney, Australia). As data were not normally distributed, descriptive statistics were calculated using medians and interquartile range for discrete and continuous variables, and frequencies and percentages for categorical variables.

To evaluate the impact of the intervention on children achieving 15 min/day of MVPA in before school care and 30 min/day of MVPA in after school care (primary outcome), a dichotomous variable was created using child MVPA minutes during each observation visit. Children were included in analyses if they had valid accelerometry data on at least one observation day at baseline or follow up. This was analysed using mixed effect logistic regression with an interaction between time × treatment. The model was clustered by service, visit, and child; and included sex, school grade and accelerometer wear time as covariates. This model was also run separately for boys and girls, without the sex covariate. Effect sizes (Cohen's *d*) were then calculated in R (version 4.3.2 R Foundation for Statistical Computing, Vienna, Austria) using the ‘esc’ (version 0.5.1) package. This calculation used the odds ratio and standard error from the logistic regression model. Effect sizes of approximately 0.2, 0.5 and 0.8 are considered small, medium, and large, respectively [35].

Mixed effect linear regression with an interaction between time × treatment was used to analyse the impact of the intervention on children's proportion of accelerometer wear time spent sedentary and in MVPA. This model was clustered by service, visit and child; and included sex and school grade as covariates. Separate models were also run on boys and girls, excluding the sex covariate. Analyses were conducted on an intention to treat basis. A statistician was consulted during the statistical analyses.

## 3 Results

### 3.1 Participants

**FIGURE 1:**
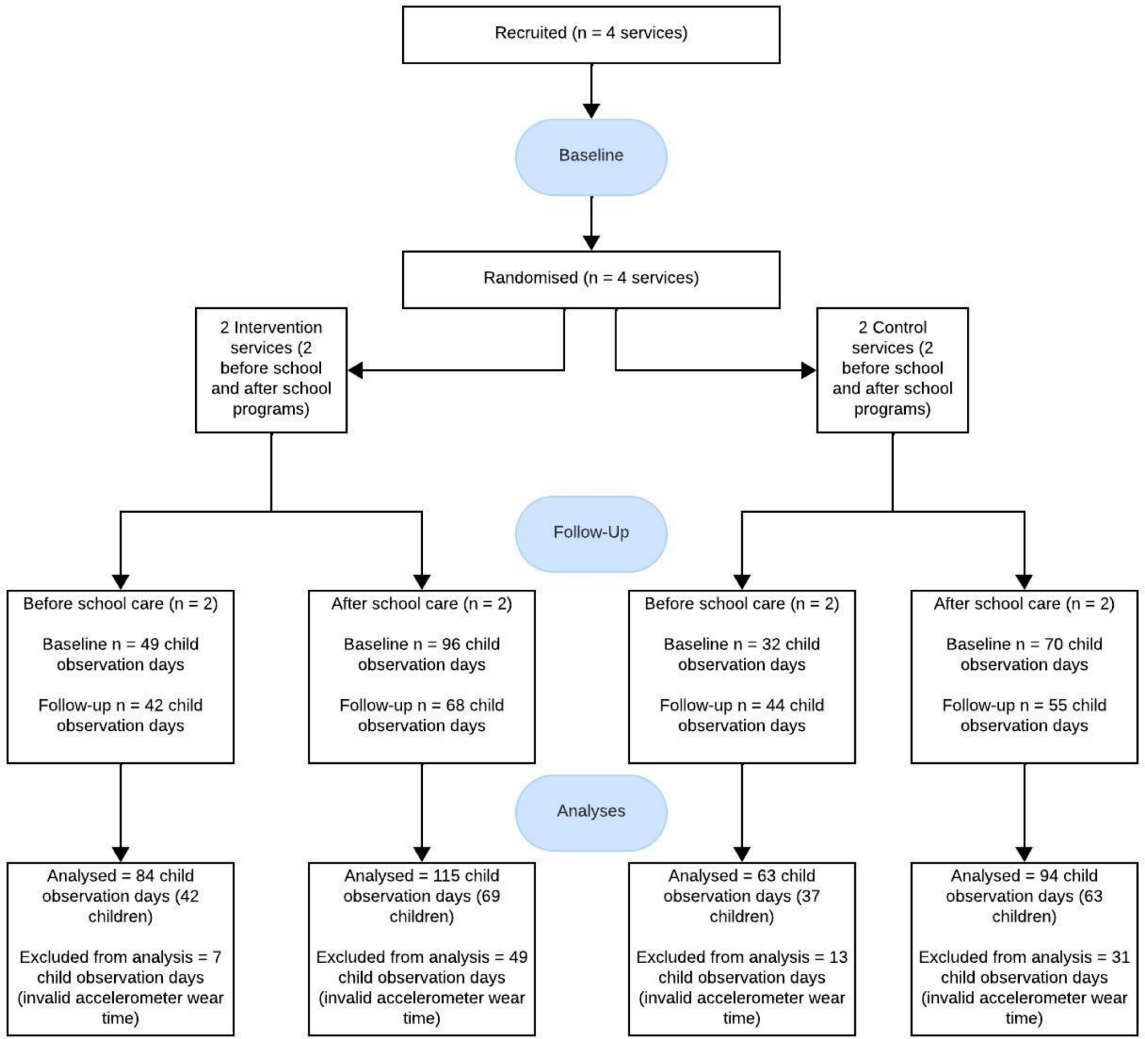
Study flow diagram.

A total of eight before school care observation visits and eight after school care observation visits were conducted across the four services involved in the study (each programme visited twice at baseline and follow up). Valid accelerometry data were analysed from 147 child observation days (79 children) in before school care, and 209 child observation days (132 children) in after school care (Figure 1). This represents data from between 35.8% and 63.8% of children in attendance during before school care observations, and between 26.8% and 39.5% during after school care observations. Baseline and follow‐up characteristics of the before and after school care programmes and children are presented in Table 1.

**TABLE 1:** Characteristics of services and objectively measured physical activity levels.

Characteristics	Before school care				After school care			
	Intervention (*n* = 2)		Control (*n* = 2)		Intervention (*n* = 2)		Control (*n* = 2)	
	Baseline	Follow‐Up	Baseline	Follow‐Up	Baseline	Follow‐Up	Baseline	Follow‐Up
Service								
Daily program length (min/day, *M*, SD)	130 (14.1)	NC	113 (10.6)	NC	180 (0.0)	NC	180 (0.0)	NC
Staff (observed during visits)								
Total	10	10	12	12	21	16	22	23
Male (%)	10	0	16.7	8.3	19	6	22.7	21.7

Children assessed via accelerometry	Boys/Girls	Total	Boys/Girls	Total	Boys/Girls	Total	Boys/Girls	Total	Boys/Girls	Total	Boys/Girls	Total	Boys/Girls	Total	Boys/Girls	Total
Number of children wearing accelerometer	23/26	49	15/27	42	11/21	32	16/28	44	46/50	96	36/32	68	36/34	70	24/31	55
Percentage of children in attendance	—	69.0	—	68.9	—	41.6	—	46.3	—	55.8	—	40.2	—	41.4	—	33.5
Number of children meeting accelerometer inclusion criteria	23/22	45	13/26	39	9/20	29	13/21	34	37/31	68	25/22	47	24/26	50	20/24	44
Percentage of children in attendance	—	63.4	—	63.9	—	37.7	—	35.8	—	39.5	—	27.8	—	29.6	—	26.8
School Grade K‐2	12/15	27	9/12	21	4/14	18	7/13	20	26/19	45	18/16	34	18/14	32	13/10	23
School Grade 3‐6	11/7	18	4/14	18	5/6	11	6/8	14	11/12	23	7/6	13	6/12	18	7/14	21
Valid accelerometer wear time (minutes/visit, Mdn, IQR)	83.0 (60.5, 102)/57.0 (45.8, 68.0)	62.0 (51.0, 93.0)	73.0 (57.0, 95.0)/53.5 (44.8, 71.3)	57.0 (48.5, 78.5)	75.0 (56.0, 84.0)/55.0 (42.8, 82.8)	61.0 (47.0, 84.0)	79.0 (68.0, 87.0)/58 (44.0, 83.0)	68.5 (52.0, 86.0)	108.0 (87.0, 118.0)/102.0 (76.0, 114.0)	105.5 (78.0, 116.0)	122 (84.0, 138.0)/95.5 (77.8, 114.0)	112.0 (82.0, 133.0)	102.5 (75.5, 122.0)/90.5 (77.0, 108.0)	91.0 (77.0, 116.0)	119.0 (101.5, 132)/98.0 (89.0, 128.0)	108.5 (93.5, 130.0)
*Physical activity levels (% of accelerometer wear time/visit, Mdn, IQR)*																
Sedentary	38.6 (29.6, 46.2)/49.7 (39.6, 58.9)	42.9 (34.5, 53.7)	43.3 (35.9, 48.3)/45.9 (29.8, 58.7)	44.2 (29.8, 55.3)	37.1 (18.3, 42.5)/42.1 (31.4, 54.9)	40.0 (29.5, 53.4)	25.6 (21.0, 42.1)/38.1 (26.7, 48.9)	31.5 (22.1, 42.3)	25.9 (18.2, 31.1)/32.2 (25.3, 47.4)	27.6 (20.9, 37.7)	15.5 (11.0, 26.5)/28.6 (21.0, 43.1)	20.9 (13.8, 37.1)	32.3 (25.1, 41.8)/35.6 (26.2, 40.5)	33.7 (26.1, 41.0)	26.5 (20.6, 34.6)/28.9 (23.8, 38.1)	28.6 (21.7, 26.3)
Light physical activity	48.9 (38.3, 54.4)/43.9 (37.9, 47.3)	44.9 (38.2, 52.4)	38.0 (36.4, 41.4)/43.7 (33.4, 53.2)	40.7 (35.7, 51.9)	40.5 (37.5, 57.0)/44.7 (37.0, 52.3)	44.7 (37.5, 53.2)	54.0 (44.0, 57.7)/41.2 (37.5, 51.7)	45.8 (39.6, 54.7)	46.3 (41.3, 50.1)/44.8 (39.8, 51.4)	45.5 (40.1, 51.2)	45.3 (37.8, 52.0)/48.2 (45.3, 55.3)	47.1 (43.3, 52.4)	48.4 (42.5, 56.1)/46.1 (41.8, 53.1)	46.7 (41.8, 54.7)	49.3 (41.8, 52.5)/45.5 (42.3, 53.6)	47.0 (41.9, 52.9)
Moderate physical activity	8.9 (5.9, 11.5)/4.4 (1.5, 7.2)	6.9 (3.5, 10.6)	8.9 (7.0, 16.0)/7.3 (3.4, 12.0)	8.2 (5.2, 12.9)	9.8 (9.5, 14.2)/8.3 (4.6, 10.9)	9.0 (5.3, 11.8)	13.2 (9.4, 14.3)/10.8 (7.2, 15.0)	12.2 (7.5, 14.8)	17.7 (11.5, 21.2)/11.4 (7.0, 14.1)	14.0 (8.7, 19.3)	23.3 (17.8, 26.5)/11.0 (6.2, 16.3)	17.8 (10.3, 24.2)	9.0 (5.7, 15.7)/10.0 (5.0, 15.0)	10.0 (5.0, 15.7)	15.1 (10.0, 21.2)/14.0 (9.7, 18.3)	14.1 (9.7, 19.6)
Vigorous physical activity	4.0 (2.6, 6.3)/1.4 (0.6, 4.8)	2.7 (1.0, 5.7)	5.6 (2.4, 8.0)/2.4 (1.4, 5.9)	3.0 (1.6, 7.5)	4.5 (2.7, 7.7)/4.2 (1.7, 6.6)	4.3 (1.7, 7.5)	4.5 (3.3, 7.5)/4.6 (2.0, 10.4)	4.6 (2.4, 10.0)	8.6 (4.3, 13.5)/4.7 (3.1, 9.2)	6.5 (3.7, 12.4)	11.3 (7.0, 18.2)/5.3 (1.5, 11.3)	8.8 (4.3, 15.4)	3.9 (1.8, 6.6)/4.4 (2.2, 10.4)	4.3 (1.9, 8.5)	10.4 (4.3, 11.3)/7.2 (4.3, 13.9)	9.4 (4.3, 12.4)
Moderate‐to‐vigorous physical activity	14.1 (9.8, 19.6)/6.3 (2.5, 12.4)	11.5 (4.7, 15.8)	15.9 (10.4, 24.0)/10.0 (5.0, 19.2)	10.7 (7.2, 20.0)	13.4 (11.5, 25.0)/12.5 (6.1, 16.9)	13.4 (8.2, 19.0)	18.8 (13.6, 24.1)/15.3 (8.6, 2.9)	17.9 (10.7, 24.8)	27.6 (17.5, 35.5)/15.9 (10.8, 21.8)	21.4 (13.1, 31.2)	34.7 (27.1, 44.6)/15.7 (9.0, 30.1)	29.8 (13.3, 37.4)	13.2 (7.8, 23.9)/14.4 (7.6, 26.0)	13.8 (7.3, 26.0)	25.0 (13.6, 35.7)/24.1 (14.4, 28.3)	24.5 (14.0, 33.5)

Abbreviations: *M* = mean; Mdn = median; NC = no change; IQR = interquartile range.

### 3.2 Intervention Feasibility and Adherence

Of the 176 OSHC services invited via email to participate in the study, four signed up to the study and provided consent. No response or acknowledgement of the study invitation was received from 135 services (77%), with nine services formally declining the invitation. Reasons for declining included being too busy to participate at this time (*n* = 5) and not being interested in the study (*n* = 4).

It was hypothesised that there would be high attendance and adherence to the professional development programme among OSHC staff in the intervention group. A total of 10 OSHC staff from the two intervention services provided consent to participate in the professional development. Of these, eight (80%) attended a professional development training and received 4 weeks of ongoing support. The reasons given for non‐attendance were unforeseen circumstances, which resulted in participants being busy on the day of training. These eight staff represent 60% of the total staff employed by the intervention services.

One intervention service had all four of their once‐weekly director phone calls completed, with the other service completing three (75%). One of the latter's weekly phone calls was not answered after three attempts across the week. In all calls conducted, service directors reported that they were working towards the goals in their action plans and their staff were regularly accessing the app.

### 3.3 Intervention Acceptability

It was hypothesised that the professional development programme would be highly acceptable to OSHC staff in the intervention group. Staff participant responses to the two feedback surveys are presented in Table 2. Eight (100%) participants either agreed or strongly agreed that the three‐hour training sessionwas enjoyable and well‐paced, improved their understanding of PA and healthy eating, taught them new skills to promote these behaviours, left them feeling motivated and facilitated the development of an achievable service action plan. Seven (88%) participants either agreed or strongly agreed that they felt supported throughout the intervention to promote PA and healthy eating in their service. Five (63%) participants either agreed or strongly agreed that the text messages were helpful. One participant reported not using the app due to issues with downloading it to their device. Of the seven participants who used the app, they all (100%) either agreed or strongly agreed that it was a useful resource. When prompted for their favourite section/s of the app, six (86%) participants selected recipes and/or activities, with menu planning, policies, and resources selected by two (29%) participants. Both service directors (*n* = 2) who received weekly phone calls strongly agreed that they were helpful.

**TABLE 2:** Professional development self‐reported satisfaction.

	Strongly agree	Agree	Neither agree nor disagree	Disagree	Strongly disagree	Not applicable^a^
Training session feedback (*n* = 8)						
Attending the professional development session improved my understanding of healthy eating in the OSHC sector.	87.50%	12.50%	0%	0%	0%	0%
Attending the professional development session improved my understanding of physical activity in the OSHC sector.	100%	0%	0%	0%	0%	0%
I learnt new skills which will help me promote healthy eating and physical activity in my OSHC service.	75%	25%	0%	0%	0%	0%
I left the training feeling motivated to implement lessons learned in my OSHC service.	87.50%	12.50%	0%	0%	0%	0%
I felt like the action plan my service developed is achievable.	75%	25%	0%	0%	0%	0%
The professional development session was well organised and paced.	100%	0%	0%	0%	0%	0%
I enjoyed my time at the professional development.	87.50%	12.50%	0%	0%	0%	0%
Ongoing support feedback (*n* = 8)						
The ESPS app was a useful resource.	62.50%	25%	0%	0%	0%	12.50%
I found the text messages helpful.	50%	12.50%	37.50%	0%	0%	0%
I found the weekly phone calls helpful.	25%	0%	0%	0%	0%	75%
I felt supported to promote healthy eating and physical activity in my service.	62.50%	25%	12.50%	0%	0%	0%

Abbreviation: ESPS = eat smart play smart.

^a^ Participant was not required to answer as they did not use the app or were not the director who received phone calls.

In response to an open‐ended question asking participants what they enjoyed most about the professional development programme, learning new information was a common response (*n* = 3) with one staff member stating: ‘Learning about how much of what foods children should be eating as well as how much PA they should be getting … was very interesting’. Participants (*n* = 2) also praised the app, with one participant sharing ‘the app helped us to be more creative with our menu and activities’ and another stating they enjoyed ‘learning the new games from using the app’. Enjoyment of the programme was also a theme (*n* = 2): ‘the fun nature of the programme’ and ‘easy to understand and well explained’.

Another question asked participants if they had any suggested improvements that could be made to the programme. Most stated they did not have any suggestions or there were no changes needed (*n* = 5). One participant shared ‘it would be great if we have more face‐to‐face programmes (like this)’; while another participant felt like the app could use more ‘simplified tabs’.

During weekly ongoing support phone calls with directors, some comments around the acceptability of the intervention components were made. One service reported they were enjoying the activities in the ESPS app, particularly, the traditional Indigenous Australian games. Another service reported that the staff training session gave them the idea to start fruit and vegetable taste testing, stating 1 week they had provided the children radish and in another week nashi pears.

App usage analytics revealed that there were 13 app downloads during the intervention period. There were 123 app sessions, with the average length of a session totalling 2 min and 30 s. App usage occurred primarily on weekdays, with only 2% occurring on a weekend.

### 3.4 Physical Activity

It was hypothesised that the proportion of children obtaining ≥ 15 min of MVPA in before school care and ≥ 30 min of MVPA in after school care would increase more in intervention services compared to control services. The proportion of children participating in at least 15 min of MVPA in before school care and 30 min of MVPA in after school care increased over time in both groups (Table 3). There were no statistically significant between‐group differences for changes in meeting recommendations, or proportion of accelerometer wear time sedentary or in MVPA. The effect sizes ranged from small (children meeting 30 min MVPA guideline; *d* = 0.08) to medium (boys meeting 15 min MVPA guideline; *d* = 0.62). There were little to no differences in change of staff PA promoting behaviours (Table 4).

**TABLE 3:** Model estimated physical activity changes.

Comparison	Proportion of children achieving ≥ 15 min of moderate‐to‐vigorous physical activity				Moderate‐to‐vigorous physical activity (% of accel wear time, Mdn)			Time spent sedentary (% of accel wear time, Mdn)		
	Within group		Between group		Within group		Between group	Between group		Within group
	Baseline	Follow‐Up	OR (95% CI)	Effect size (Cohen *d*)	Baseline	Follow‐Up	Estimate (95% CI)	Baseline	Follow‐Up	Estimate (95% CI)
*Before school care*										
Total										
Intervention	8.90%	17.90%	1.91 (0.17, 21.08)^a^	0.36	11.50%	10.70%	−1.0 (−6.2, 4.2)	42.90%	44.20%	5.43 (−2.99, 13.85)
Control	13.80%	32.40%			13.40%	17.90%		40.00%	31.50%	
Boys										
Intervention	17.40%	46.20%	3.06 (0.17, 53.73)^b^	0.62	14.10%	15.90%	2.8 (−5.3, 10.8)^d^	38.60%	43.30%	8.80 (−4.88, 22.48)^d^
Control	22.20%	38.50%			13.40%	18.80%		37.10%	25.60%	
Girls										
Intervention	0.00%	3.80%	—	—	6.30%	10.00%	−3.9 (−11.2, 3.4)^d^	49.70%	45.90%	2.91 (−8.43, 14.26)^d^
Control	10.00%	28.60%			12.50%	15.30%		42.10%	38.10%	

After school care	Proportion of children achieving ≥ 30 min of moderate‐to‐vigorous physical activity									
Total										
Intervention	27.90%	42.60%	1.15 (0.223, 5.88)^a^	0.08	21.40%	29.80%	0.27 (−5.86, 6.40)^c^	27.60%	20.90%	−1.59 (−8.87, 5.69)^c^
Control	20.00%	36.40%			13.80%	24.50%		33.70%	28.60%	
Boys										
Intervention	45.90%	68.00%	0.75 (0.07, 7.59)^b^	−0.16	27.60%	34.70%	1.39 (−7.30, 10.08)^d^	25.90%	15.50%	−1.41 (−10.79, 7.98)^d^
Control	20.80%	50.00%			13.20%	25.00%		32.30%	26.50%	
Girls										
Intervention	6.45%	13.64%	2.04 (0.19, 22.21)^b^	0.39	15.90%	15.70%	−1.85 (−10.29, 6.60)^d^	32.30%	28.60%	−0.52 (−11.94,10.91)^d^
Control	19.23%	25.00%			14.40%	24.10%		35.60%	28.90%	

*Note:* Models clustered by service, visit and child.

Abbreviation: —, sample too small to model.

^a^ Covariates of sex, school grade K‐2 or 3–6 and wear time in minutes.

^b^ Covariates of grade K‐2 or 3‐6 and wear time in minutes.

^c^ Covariates of sex and school grade K‐2 or 3–6.

^d^ Covariates of school grade K‐2 or 3–6.

**TABLE 4:** Food and beverages provided and observed staff behaviour differences.

Food description	Before school care						After school care					
	Control			Intervention			Control			Intervention		
	Baseline (*n* = 4)	Follow‐Up (*n* = 4)	Difference	Baseline (*n* = 4)	Follow‐Up (*n* = 4)	Difference	Baseline (*n* = 4)	Follow‐Up (*n* = 4)	Difference	Baseline (*n* = 4)	Follow‐Up (*n* = 4)	Difference
Fruit	2	2	—	1	3	+2	4	4	—	4	4	—
Vegetables	0	0	—	0	2	+2	2	3	+1	2	4	+2
Dairy	4	4	—	4	4	—	2	2	—	1	4	+3
Grains	4	4	—	4	4	—	2	2	—	4	2	−2
Wholegrains or higher fibre	4	4	—	4	3	−1	2	2	—	3	2	−1
Refined grains or lower fibre	4	4	—	4	4	—	0	0	—	1	0	−1
Lean meats and alternatives	1	1	—	0	0	—	0	0	—	0	0	—
Discretionary foods	4	4	—	4	2	−2	4	4	—	3	2	−1
Beverages												
Water	4	4	—	4	4	—	4	4	—	4	4	—
Fruit juice	4	4	—	0	0	—	0	0	—	0	0	—
Staff behaviour												
Verbally promoting healthy eating	0	0	—	0	1	+1	0	2	+2	2	3	+1
Verbally discouraging healthy eating	0	0	—	0	0	—	0	0	—	0	0	—
Verbally promoting physical activity	2	3	+1	2	1	−1	3	4	+1	4	3	−1
Verbally discouraging physical activity	0	1	+1	1	1	—	2	3	+1	3	2	−1
Engaged in physical activity	2	3	+1	2	2	—	2	3	+1	4	4	—

*Note:* Reported as number of visits, the food group was provided or staff behaviour observed.

### 3.5 Food and Beverage Provision

This study hypothesised that the frequency of observation days fruit and vegetables were provided in before and after school care would increase more in intervention services compared to control. In before school care intervention services, the number of observation days vegetables were provided increased at follow‐up from 0% to 50% (*n* = 2) and fruit from 25% (*n* = 1) to 75% (*n* = 3) (Table 4). There was no change in control services with fruit and vegetables provided on 0% of days. In after school care, the number of observation days vegetables were provided increased at follow up from 50% (*n* = 2) to 100% (*n* = 4) in intervention services, with a change of 50% (*n* = 2) to 75% (*n* = 3) of days in control services. There was no change in after school care fruit provision, remaining at 100% (*n* = 4) in both intervention and control services. There were little to no differences in the change of staff nutrition promoting behaviours.

## 4 Discussion

This pilot RCT, to the author's knowledge, is the first known published study to evaluate the impact a combined PA and healthy eating staff professional development programme can have on objectively measured child PA and food provision in Australian OSHC services. An important contribution from this research was the strong feasibility and acceptability of our multifaceted intervention among study participants. OSHC services have a high staff turnover and consist largely of casual staff [9]. Therefore, achieving an attendance of nearly 60% from the total staff employed by intervention services participating in the professional development has been identified as a good turnout. No monetary incentives were offered to service providers; thus, they would likely have paid their own staff for attendance at the 3 h training session. Their willingness to do this for a large proportion of their staff demonstrates its perceived value to the services.

Additionally, staff responses to the two feedback surveys were very positive, with participants agreeing and strongly agreeing that the intervention increased their understanding and skills to promote PA and healthy eating, and feeling supported to do so. This aligns with a recent survey of Australian OSHC services which highlighted staff training and educator understanding as the most important enablers for implementing PA guidelines in the setting [36]. While the ESPS app and weekly director phone calls were viewed as useful by all participants, some participants neither agreed nor disagreed that the text messages were helpful. The content of these messages was from a pre‐written bank of health‐promoting messages developed by the research team, with appropriate ones selected based on the service's action plan goal. Future trials incorporating text messages for OSHC staff should consider options to make these messages more engaging, such as clickable links to resources which have seen success in parental health promotion studies [37].

In considering these feasibility and acceptability outcomes, several implications should be acknowledged. First, while there was high participation in the programme from staff in intervention services, challenges were faced recruiting OSHC services to participate in the study. With no response to study invitation emails received from 77% of invited services, future trials and scale‐up of similar programmes in OSHC should employ additional recruitment methods. Recruitment phone calls were made to some services; however, many unanswered calls and requests to resend the recruitment email may suggest constraints in staff time. Monetary incentives for participants have been employed by OSHC interventions previously [24] and may help with recruitment; however, this is not feasible for a full‐scale efficacy or effectiveness trial. Working more closely with service providers who manage multiple services and including more resources in the recruitment stage has been successful in family childcare interventions [38] and could be considered in OSHC settings.

Additionally, while the participating OSHC services were happy for their staff to attend the 3‐h training session, this may not be applicable to all services. The staff time costs could have been a deterrent during service recruitment, with services potentially not seeing the value for money or not being able to afford it. While the co‐creation stakeholders indicated a preference for in‐person training, and studies have found OSHC interventions to be more effective when delivered this way [24], offering an online option could save costs and increase uptake of the programme. A train‐the‐trainer model could also be considered and would require less staff from a service needing to attend the session. Future trials should consider different mediums of delivery and conduct a cost analysis of the programme.

Another strength of this intervention was its utilisation of a co‐creation framework [21] and involvement of various stakeholders and end‐users. Having end‐users involved in public health intervention co‐creation is widely understood to result in more effective programmes [39] and lead to increased consumer satisfaction and adherence [40]. This was reflected in the positive feasibility and acceptability outcomes discussed above, with the co‐creation process likely contributing to this. While not conducted in this study, a recommendation for future co‐creation work of this nature would be to hold an additional focus group with co‐creators after completion of intervention testing. This would allow reflection on any successes and limitations of the intervention and provide co‐creators an opportunity to contribute to further refinement of the intervention for any future up‐scaling.

No between group differences for PA outcomes were statistically significant; however, small effect sizes were detected for total children in before school care (*d* = 0.36) and girls in after school care (*d* = 0.39) meeting the 15 and 30 min of MVPA guidelines, respectively. A moderate effect size was also detected for boys in before school care (*d* = 0.62) meeting the 15 min guideline. This suggests the potential efficacy of the professional development programme. A larger sample size from a fully powered RCT would assist in confirming this finding. Increasing the number of children meeting these guidelines is important as it will assist children in adhering to the MVPA component of the 24‐h movement behaviour guidelines (60 min per day) which is associated with improved health outcomes for children [41].

The intervention also had a positive change on the food that services were providing, with before school care intervention programmes increasing the number of days they offer both fruit and vegetables, compared to no change seen in control services. After school care intervention programmes also increased the days they provided vegetables at a larger margin than control programmes. This is a promising finding as a US OSHC study found children often consume fruits and vegetables when they are provided by the service [42], so increased provision from this intervention could assist children to meet nutritional guidelines. However, due to the small sample size of this pilot study (only observing food provision in four services), this finding cannot demonstrate efficacy of the intervention. Larger trials of the impact staff professional development can have on fruit and vegetable provision in OSHC are required.

Regarding the study's secondary potential efficacy outcomes of improved staff health promoting behaviours, there was little change postintervention. The lack of change in after school care PA promotion can be attributed to it already being observed on 100% of visits at baseline; however, there were areas for improvement in before school care and in nutrition promotion across both settings. A potential reason for this could be the small sample size of two intervention services not providing enough observation visits to see behaviour change. Regardless, a stronger emphasis on the importance of staff verbally promoting healthy eating and PA and engaging in play with children should be added to future health promotion programmes and professional development initiatives. These behaviours have been associated with child activity in OSHC [43] and food consumption in other childcare settings [44]. It is also important to note that the staff behaviours reported in this study are not the only ones that can influence healthy behaviours in children. Factors such as activity context and equipment availability, which are influenced by staff, can also contribute to child PA in OSHC [13, 14]. These behaviours were not outcomes of this study; however, future intervention studies could benefit from reporting how factors such as these are influenced by professional development.

This study contributes towards the slowly growing evidence around the importance of the OSHC sector for promoting PA and healthy eating in children aged 5–12 years. A review by Sliwa et al. [45] highlighted that OSHC programmes can complement school‐day efforts to address PA and healthy eating as an additional opportunity in the extended day. It also found that organisation‐level policy and programmatic interventions can support youth behaviours in these settings; also reinforced in a review of OSHC PA interventions [46]. The results of this study demonstrate how staff professional development around PA and healthy eating has high feasibility and acceptability within the Australian OSHC context. Further large‐scale studies are needed to confirm how meaningful an impact such interventions can have on the healthy eating environment of services and PA levels of children; however, the findings of this study indicate potential.

### 4.1 Strengths and Limitations

This study has several strengths and limitations. Strengths include: (1) using a co‐design framework for intervention development; (2) collecting device‐measured PA and (3) directly observing staff behaviours and food provision. Limitations which should be considered include: (1) a small sample size limiting statistical power; (2) a short intervention and follow‐up period which could both reduce the opportunity for changes to occur and not observe longer term impacts; (3) vacation care was not a focus of this study so findings cannot be generalised across all OSHC programmes and (4) although the first staff feedback survey was anonymous and conducted online, there is the potential for social desirability bias from it being completed in an environment where the researcher was present.

## 5 Conclusions

To conclude, a PA and healthy eating multifaceted professional development intervention in OSHC has high feasibility and acceptability. It had a positive impact on fruit and vegetable provision in before school care and vegetable provision in after school care; however, larger trials are required to demonstrate efficacy on food outcomes. While there were no statistically significant impacts on meeting MVPA guidelines or activity levels in this pilot study, small to moderate effect sizes warrant future studies with larger sample sizes to evaluate intervention efficacy and effectiveness. Longer intervention and follow‐up periods could also allow the long‐term effects of the programme and policy change to be observed. Furthermore, a cost analysis of the programme should be conducted and alternative mediums of delivery explored to support real world application and scaling up the programme.

## Ethics Statement

The intervention co‐creation was approved by the University of Wollongong Human Research Ethics Committee (2021/ETH11747). The pilot randomised controlled trial was approved by the University of Wollongong Human Research Ethics Committee (2023/ETH00140) and registered prospectively with the Australian New Zealand Clinical Trials Registry (ACTRN12623000176662p).

## Conflicts of Interest

The authors declare no conflicts of interest.

## Supporting information


**Data S1.** Supporting Information.

**Data S2.** Supporting Information.

**Data S3.** Supporting Information.

**Data S4.** Supporting Information.

## Data Availability

The data that support the findings of this study are available on request from the corresponding author. The data are not publicly available due to privacy or ethical restrictions.
