# Supplementary material for: Development and Testing of an Out‐of‐School Hours Care Professional Development Program: A Pilot Cluster Randomised Controlled Trial
Source: Health Promot J Austr. 2025 Jun 10;36(3):e70056. doi: 10.1002/hpja.70056 (PMC12150000; doi:10.1002/hpja.70056)
Supplement: Supplementary file 1 — Data S1. Supporting Information. [file HPJA-36-0-s002.docx]

**Supporting Information - Intervention Development**

*Planning*

According to Leask et al. ^1^, the two main principles of planning are framing the aim of the study and identifying an appropriate sampling strategy. The aim of co-creation was to involve OSHC stakeholders and end-users in the co-creation of an intervention for the promotion of physical activity (PA) and healthy eating in OSHC services. This was to be achieved through two online focus groups (and semi-structured interviews where participants could not attend scheduled focus groups); one in-person focus group with children who attend OSHC; and one in-person session to deliver the intervention to co-creators and obtain final input. Focus groups are common to participatory methodology in the childcare space ^2^ and allow for collaboration among co-creators.

To ensure an appropriate sampling strategy, a plan to seek representation from different stakeholders and end-users was made. Co-creators who were invited to attend the two online focus groups included OSHC industry representatives, service directors, and service educators. They were recruited via a convenience sample, with participants involved in previous research conducted by the researchers ^3^ invited through email to participate. Children were recruited from a service participating in the co-creation focus groups and invited to be involved in a single in-person focus group occurring at an OSHC service. University and Government stakeholders were involved through a pre-existing working group to oversee the project that this research is part of. This ENHANCE OSHC working group met monthly and consisted of members from the Illawarra Shoalhaven Local Health District; South Western Sydney Local Health District; NSW Centre for Population Health; and the University of Wollongong, Australia.

*Conducting*

The conducting stage involves manifesting ownership and defining the procedure ^1^. Ownership of the developed intervention was built among co-creators by emphasising that there was no pre-planned intervention, and their input would inform the design process. The participants were involved at every stage of development to maintain their ownership.

There were six co-creators (one OSHC industry representative, three service directors and two service educators) who participated in the online focus groups, and twelve children from one service who attended the in-person group. Each focus group was 60 minutes, with the final in-person session to deliver the intervention to co-creators running for three hours. To meet the aim of intervention co-creation, the topics of the focus groups were as follows:

1. Online focus group one – key findings were presented from previous before and after school care audits ^3–5^ and barriers and facilitators of PA and healthy eating in the OSHC sector were explored. This assisted with upskilling and empowering co-creators ^1^.Attendees were asked to reflect on the research findings and share their personal experiences and insights of these environments in OSHC.
2. In-person child focus group – attitudes towards the activities and foods children liked in OSHC were explored to strengthen end-user perspectives of the co-creation process. Discussion questions were phrased in an age-appropriate way with the support of members of the research team with extensive experience conducting child research. Large sheets of paper and markers were used to allow children to share their ideas creatively through words and pictures. A staff member of the OSHC service was present during the session to help the children feel comfortable.
3. Online focus group two – possible intervention options in the OSHC setting were discussed and participants were asked what they thought could address the barriers identified in the first online focus group. Findings from the child focus group were also shared to provide co-creators with the child perspective. The outcome of this focus group was co-creators indicating a preference for staff professional development as the intervention mode of delivery.
4. In-person intervention presentation session – a draft staff professional development program was developed using the discussion points and feedback from the focus groups; and informed by health education content and resources from several sources including the Eat Smart Play Smart manual ^6^, the Australian Government’s Eat for Health Educator Guide ^7^, and the Victorian Governments Healthy Eating Advisory Service OSHC resources ^8^. Co-creators were invited to attend a session where the 3-hour training component of the program was delivered, and the other components of the draft program shared. At the end of the session attendees were asked to share their final thoughts and feedback on the program.

Following the focus groups and intervention session, the co-created intervention was refined and was provided to the University and Government stakeholders for their feedback. This multi-step process ensured the intervention was informed by the population it aimed to support and involved varying perspectives.

*Evaluating*

The evaluation consisted of two parts, the evaluation of the co-creation process and the evaluation of the intervention. Evaluation of the co-creation was embedded throughout the process. Member checking ^9^ occurred in each online focus group, where the results from the previous session were reviewed. Respondent validation ^10^ was used, and participants were presented with the intervention in the final focus group. Evaluation of the developed intervention occurred through a pilot randomised controlled trial (RCT), presented in this study.

*Reporting*

Reporting involves explaining the co-creation process in a way that is understood, reproducible and systematic. The checklist for reporting intervention co-creation proposed by Leask et al. ^1^ was used.

**References**

1 Leask CF, Sandlund M, Skelton DA, Altenburg TM, Cardon G, Chinapaw MJM *et al.* Framework, principles and recommendations for utilising participatory methodologies in the co-creation and evaluation of public health interventions. *Res Involv Engagem* 2019; **5**: 2.

2 Adler K, Salanterä S, Zumstein-Shaha M. Focus Group Interviews in Child, Youth, and Parent Research: An Integrative Literature Review. *Int J Qual Methods* 2019; **18**. doi:10.1177/1609406919887274/ASSET/IMAGES/LARGE/10.1177_1609406919887274-FIG1.JPEG.

3 Woods AJ, Norman J, Ryan ST, Wardle K, Probst YC, Crowe RK *et al.* Children’s physical activity and sedentary behaviour in before school care: An observational study. *Prev Med* 2024; **178**: 107810.

4 Crowe RK, Probst YC, Norman JA, Furber SE, Stanley RM, Ryan ST *et al.* Foods and beverages provided in out of school hours care services: an observational study. *BMC Public Health* 2022; **22**. doi:10.1186/s12889-022-12652-9.

5 Crowe RK, Probst YC, Stanley RM, Ryan ST, Weaver RG, Beets MW *et al.* Physical activity in out of school hours care: an observational study. *International Journal of Behavioral Nutrition and Physical Activity* 2021; **18**: 1–8.

6 National Heart Foundation of Australia. *Eat Smart Play Smart - A Manual for Out of School Hours Care*. Third. 2016.

7 National Health and Medical Research Council. Eat For Health Educator Guide. 2013.https://www.eatforhealth.gov.au/sites/default/files/2022-09/n55b_educator_guide_140321_1.pdf (accessed 25 Sep2023).

8 Healthy Eating Advisory Service. Outside school hours care (OSHC). https://heas.health.vic.gov.au/im-working-in/outside-school-hours-care/ (accessed 9 Feb2024).

9 Carlson JA. Avoiding Traps in Member Checking. *The Qualitative Report* 2010; **15**: 1102–1113.

10 Silverman D. *Doing Qualitative Research*. 5th Edition. Sage Publications, 2017.
