# Supplementary material for: Development and Testing of an Out‐of‐School Hours Care Professional Development Program: A Pilot Cluster Randomised Controlled Trial
Source: Health Promot J Austr. 2025 Jun 10;36(3):e70056. doi: 10.1002/hpja.70056 (PMC12150000; doi:10.1002/hpja.70056)
Supplement: Supplementary file 2 — Data S2. Supporting Information. [file HPJA-36-0-s001.pdf]

# ENHANCE OSHC Professional Development Session Structure

**9:50** – Participant registration.

**10:00** – Welcome and session overview. Participants will be asked to download the Eat Smart Play Smart app.

**10:10** – Icebreaker

**10:20** – Presentation on nutrition in OSHC findings and areas for potential improvement.

**10:40** – Nutrition promotion skill development activities (case study; group brainstorming; ESPS resources).

**11:00** – Nutrition policy discussion.

**11:15** – Service nutrition goal setting.

**11:30** – Morning tea break.

**11:45** – Presentation on physical activity (PA) in OSHC findings and areas for potential improvement.

**12:00** – PA promotion skill development activities (practical using the ESPS app activities).

**12:20** – Embedding PA targets into service programming.

**12:35** – Service PA goal setting.

**12:50** – Ongoing support discussion and conclusion.

**13:00** – Session end.
