# Supplementary material for: Development and Testing of an Out‐of‐School Hours Care Professional Development Program: A Pilot Cluster Randomised Controlled Trial
Source: Health Promot J Austr. 2025 Jun 10;36(3):e70056. doi: 10.1002/hpja.70056 (PMC12150000; doi:10.1002/hpja.70056)
Supplement: Supplementary file 4 — Data S4. Supporting Information. [file HPJA-36-0-s004.pdf]

4-Week Action Plan - Healthy Eating

Service Name: ___________________________________

Break down your mission into 3 SMART goals:

Goal 1	Action Steps:
	______________________
	______________________
	______________________
	______________________

Goal 2	Action Steps:
	______________________
	______________________
	______________________
	______________________

Goal 3	Action Steps:
	______________________
	______________________
	______________________
	______________________

Signed: ___________________________
